# Anti-Microbial Activity of Aliphatic Alcohols from Chinese Black Cardamom (*Amomum tsao*-*ko*) against *Mycobacterium tuberculosis* H37Rv

**DOI:** 10.3390/plants12010034

**Published:** 2022-12-21

**Authors:** So Young Lee, Gauri S. Shetye, So-Ri Son, Hyun Lee, Larry L. Klein, Jeffrey K. Yoshihara, Rui Ma, Scott G. Franzblau, Sanghyun Cho, Dae Sik Jang

**Affiliations:** 1 Department of Biomedical and Pharmaceutical Sciences, Graduate School, Kyung Hee University, Seoul 02447, Republic of Korea; 2 Institute for Tuberculosis Research, Department of Pharmaceutical Sciences, College of Pharmacy, University of Illinois at Chicago, Chicago, IL 60612, USA; 3 Biophysics Core at Research Resource Center, University of Illinois at Chicago, 1100 S. Ashland Ave, Chicago, IL 60607, USA; 4 Department of Pharmaceutical Science, College of Pharmacy, Kyung Hee University, Seoul 02447, Republic of Korea

**Keywords:** *Amomum tsao*-*ko*, Zingiberaceae, aliphatic alcohols, *Mycobacterium tuberculosis*, anti-tubercular activity

## Abstract

The fruits of *Amomun tsao-ko* (Chinese black cardamom; Zingiberaceae) contain an abundance of essential oils, which have previously demonstrated significant antimicrobial activity. In our preliminary search for natural anti-tuberculosis agents, an acetone extract of *A*. *tsao*-*ko* (AAE) exhibited strong antibacterial activity against *Mycobacterium tuberculosis* H37Rv. Therefore, the aim of this study was to find the principal compounds in an AAE against *M*. *tuberculosis*. Nine aliphatic compounds (**1**–**9**) including a new compound (**1**, tsaokol B) and a new natural unsaturated aliphatic diester (**6**), together with three acyclic terpenoids (**10**–**12**), were isolated from an AAE by repetitive chromatography. The structures of the isolates were determined by spectroscopic data analysis. All isolates were evaluated for activity against *M*. *tuberculosis* H37Rv. Isolated compounds **1**–**6**, and **11** had MICs ranging from 0.6–89 µg/mL. In contrast, compounds **7** to **10**, and **12** had MICs that were >100 µg/mL. Tsaokol A (**3**) was the most active compound with MICs of 0.6 µg/mL and 1.4 µg/mL, respectively, against replicating and nonreplicating *M*. *tuberculosis*. These results are the first to illustrate the potency of tsaokol A (**3**) as a natural drug candidate with good selectivity for treating tuberculosis.

## 1. Introduction

Throughout recorded history, spices and aromatic herbs have been used worldwide in cooking to increase the flavor of food due to their characteristic taste and aroma. According to the International Organization for Standardization (ISO), there are 109 types of spices belonging to 31 families, and these spices contain essential oils that give them their own distinctive aroma [1,2]. Essential oils consist of various specialized metabolites (mainly composed of terpenoids, aromatic compounds, and aliphatic compounds) that are synthesized by aromatic plant families: Myrtaceae, Lauraceae, Rutaceae, Lamiaceae, Asteraceae, Apiaceae, Cupressaceae, Poaceae, Piperaceae, and Zingiberaceae [3]. While essential oils have been typically utilized as flavoring ingredients, they have also been used in traditional medicine [4]. A recent study demonstrated that essential oils possess strong antibacterial activity against Gram-positive bacteria, and that their antibacterial mechanism widely differs, depending on their chemical composition [4].

The family Zingiberaceae is a well-known aromatic plant family that has been considered a rich source of essential oils. Particularly, the fruits of *Amomum tsao*-*ko* Crevost et Lemaire (Chinese black cardamom; Zingiberaceae) have an abundance of essential oils with a distinctive aroma. Due to its unique fragrance, *A*. *tsao-ko* is one of the significant spices that is extensively used in southeast Asia and China. In addition to its role in enhancing flavor, it has been used in traditional oriental medicine for the treatment of ailments, such as gastrointestinal disorders, liver abscesses, and throat infections [5,6]. In previous studies, essential oils of *A*. *tsao*-*ko* have shown strong antibacterial activity against food borne bacteria or fungi, especially the *Staphylococcus aureus* species [7]. However, studies on the antimicrobial activity of the *A*. *tsao*-*ko* fruits on *Mycobacterium tuberculosis* have not been reported.

Tuberculosis (TB) is a major global health problem with 1.5 million deaths each year, further exacerbated by a rapid increase in the multidrug-resistant (MDR) and extensively drug-resistant (XDR) *M*. *tuberculosis* strains [8,9]. This critical need has compelled researchers to develop new drugs with possibly new cellular targets for the treatment of MDR and XDR *M*. *tuberculosis* strains [10]. Though promising leads have been reported and a few drugs have been approved in recent years, the tuberculosis drug pipeline remains sparse and far from ideal [11]. In this report, a phenotypic screening against *M. tuberculosis* H37Rv was performed to select active hits from plant extracts, which are still a potential natural product source for finding new antibiotics. In a preliminary study to identify new TB drug leads, an acetone extract from the fruits of *A*. *tsao*-*ko* (AAE) was found to show strong growth inhibitory activity against *M. tuberculosis*. Therefore, the purpose of this study is to find the hit compounds in AAE against *M*. *tuberculosis*.

Herein, repeated chromatography with the acetone extract of *Amomum tsao*-*ko* was conducted to isolate compounds (**1**–**12**). The structure of the new compound **1** was elucidated by interpreting 1D- and 2D-nuclear magnetic resonance (NMR) spectroscopic data and high-resolution dart mass (HR-DART-MS) spectrometric data. All of the isolated compounds (**1**–**12**) were evaluated for their anti-tuberculosis effect against *M*. *tuberculosis* H37Rv. The isolation of compounds from AAE and the anti-tubercular activity of the isolates are described below.

## 2. Results and Discussion

### 2.1. Structure Elucidation of Compound ***1*** and Identification of ***2***–***12***

**Figure 1:**
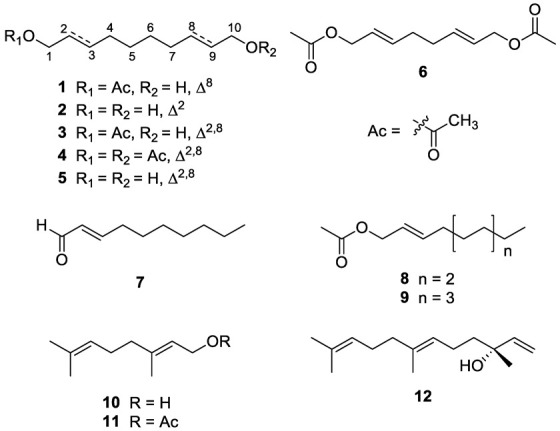
Chemical structures of compounds **1**–**12** isolated from the fruits of *A*. *tsao-ko*.

In the present study, nine aliphatic compounds (**1**–**9**) including a new isolate (**1**) and a new natural (**6**) unsaturated aliphatic diester, together with three acyclic terpenoids (**10**–**12**), were isolated from the fruits of *A*. *tsao*-*ko* by repetitive chromatography (Figure 1).

**Figure 2:**
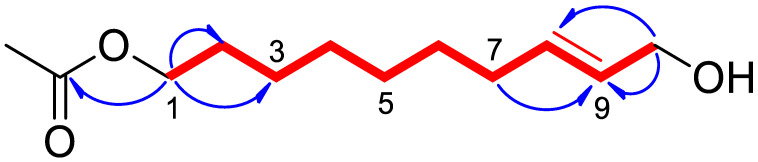
Selected correlations observed in the COSY (▬) and HMBC (→) NMR spectra of compound **1**.

Compound **1** was obtained as a colorless oil, and its molecular formula was determined as C_12_H_22_O_3_ by HR-DART-MS (*m*/*z* 232.1900 [M+NH_4_]^+^; calcd for C_12_H_26_N_1_O_3_ 232.1913) (Figure S1). The presence of O-H (3437 cm^−1^), C-H (2921 and 2851 cm^−1^), C=O (1737 cm^−1^), and C-O (1236 cm^−1^) groups in compound **1** was revealed by the infrared (IR) spectrum (Figure S2). The ^1^H NMR of compound **1** showed two *trans*-olefinic [*δ*_H_ 5.67 (1H, dt, *J* = 15.5, 6.5 Hz), 5.61 (1H, dt, *J* = 15.5, 5.5 Hz)], two oxygenated methylene [*δ*_H_ 4.03 (2H, t, *J* = 7.0 Hz), 4.07 (2H, d, *J* = 5.5 Hz)], and one methyl [*δ*_H_ 2.02 (3H, s)] signals (Table 1 and Figure S3). Additionally, six characteristic methylene resonances [*δ*_H_ 2.02 (2H, m), 1.59 (2H, m), 1.20–1.39 (8H, m)] of the aliphatic chain were observed in the ^1^H NMR spectrum. The ^13^C NMR and ^1^H-^13^C heteronuclear single quantum coherence spectroscopy (HSQC) spectra revealed the presence of a carbonyl (*δ*_c_ 171.5), a methyl (*δ*_c_ 21.2), two olefinic methine (*δ*_c_ 133.6 and 129.1), two oxygenated methylene (*δ*_c_ 64.8 and 64.1), and six methylene (*δ*_c_ 26.0, 29.2, 29.3, 28.7, 29.9, and 32.4) carbons in compound **1** (Table 1, Figures S4 and S5). In further analysis with the ^1^H-^1^H coherence spectroscopy (COSY) and ^1^H-^13^C heteronuclear multiple bond correlation (HMBC) data, it was inferred that compound **1** is an unsaturated aliphatic alcohol with an acetyl moiety (Figure 1). The position of the acetyl group was established at C-1 through the observed HMBC correlations between H-1 and/OCOCH_3_,/C-2, and C-3 (Figure 2 and Figure S7). Furthermore, the position of the *trans*-olefinic group was confirmed at C-8 by the ^1^H-^1^H COSY and HMBC spectra (Figure 2, Figures S6 and S7). Compound **1** has a very similar structure to tsaokol A [12], except for the absence of one double bond at C-2 (Figure 1 and Table 1). As a result, the chemical structure of compound **1** was elucidated as (*E*)-1-acetyl-8-decene-1,10-diol and named tsaokol B.

By comparing the spectroscopic data with those reported in the literature, the known compounds were identified to be (*E*)-2-decene-1,10-diol (**2**) [13], tsaokol A [(2*E*,8*E*)-1-acetyl-2,8-decadiene-1,10-diol) (**3**) [12], acetoxytsaokol A [(2*E*,8*E*)-1,10-diacetyl-2,8-decadiene-1,10-diol] (**4**) [12], (2*E*,8*E*)-2,8-decadiene-1,10-diol (**5**) [14], (*E*)-decenal (**7**) [15], (*E*)-1-acetyl-2-decene-1-ol [(*E*)-2-decen-l-yl acetate] (**8**) [16], (*E*)-1-acetyl-2-dodecene-1-ol [(*E*)-2-dodecen-1-yl acetate] (**9**) [17], geraniol (**10**) [18], geranyl acetate (**11**) [19], and (3*R*)-(*E*)-nerolidol (**12**) [20,21]. Although compound **6** [(2*E*,6*E*)-1,8-diacetoxy-2,6-octadiene] was previously synthesized as an intermediate [22], this is the first report on the isolation of compound **6** from natural sources (Figures S8–S11).

### 2.2. Antimicrobial Activitiy of Isolated Compounds aganist M. tuberculosis H37Rv

Non-replicating persisters *M*. *tuberculosis* are partly responsible for the long duration of TB therapies [8]. In order to shorten TB treatment duration, new TB drugs would need to be effective not only against the replicating *M*. *tuberculosis*, but also against these non-replicating persisters *M*. *tuberculosis* [9]. Therefore, the experiment was carried out in a regular environment for replicating *M*. *tuberculosis* H37Rv (MABA) and in a low oxygen environment for non-replicating *M*. *tuberculosis* H37Rv (LORA). First, the activity of the AAE was confirmed against both actively growing and non-replicating *M*. *tuberculosis* (Table 2). The MABA MIC of the AAE was 9.7 µg/mL and the LORA MIC of the AAE was 86.8 µg/mL. Second, to identify the active principle(s) of the AAE, twelve compounds were isolated and tested for anti-tubercular activity. Compounds **1**, **3**, **4**, and **5** showed MICs lower than 10 µg/mL. MICs of compounds **2**, **6**, and **11** were greater than 20 µg/mL. In contrast, compounds **7**–**10** and **12** had MICs greater than 100 µg/mL (Table 2). All of the active compounds against the replicating *M*. *tuberculosis* also showed activity against non-replicating *M*. *tuberculosis* under low oxygen conditions. Finally, among all of the isolated compounds, tsaokol A (**3**) showed the lowest MICs of MABA and LORA. Notably, the LORA MIC of tsaokol A (**3**) was only two-fold higher than that of the MABA MIC (Table 2). This ratio of non-replicating MIC over replicating MIC for tasaokol A (**3**) is promising in comparison with the first line TB drugs rifampin and isoniazid (Table 2). Moreover, cytotoxicity (IC_50_) of tsaokol A (**3**) against the Vero cell line was greater than 200 µg/mL.

To validate its activity, tsaokol A (**3**) was chemically synthesized and its activity was investigated under the same conditions. The MICs and cytotoxicity data of the synthesized tsaokol A were identical to those of the isolated tsaokol A (**3**). Therefore, this report has found a potent anti-TB small molecule from a natural product with good selectivity. Except for the report of inhibition of sphingosine kinases SPHK1/2 [12], no other bioactivity had previously been described for tsaokol A (**3**). The anti-tubercular activities of compounds **1**–**5** suggest that the presence of an olefinic group at C-8 is important, though additional research is needed to clarify this structure-activity relationship. Toward that end, modification of the functional groups (olefin, ester, alcohol) and of the chemical composition (chain length, linker atoms) will be carried out in due course.

## 3. Materials and Methods

### 3.1. General Experimental Procedures

Thin layer chromatography (TLC) analyses were performed on Silica gel 60 F_254_ (Merck KGaA, Darmstadt, Germany) and RP-18 F_254S_ (Merck KGaA) plates. After TLC development in a confirmed solvent system, TLC plates were charred by 20% (*v*/*v*) H_2_SO_4_ reagent (Duksan, Seoul, Republic of Korea) and then heated at 123 °C for 10 min. The UV spectrum was obtained with a Perkin Elmer Lambda 35 UV/VIS spectrometer (PerkinElmer, Shelton, CT, USA). Optical rotations were obtained on a Jasco P-2000 polarimeter (JASCO, Tokyo, Japan), using a 10 mm microcell. JEOL (JEOL, Tokyo, Japan) 500 MHz was used for obtaining NMR spectra. HR-DART-MS spectra were obtained by the DART ion source (Ionsense, Tokyo, Japan) coupled to an AccuTOF-TLC (JEOL, Tokyo, Japan). An Agilent Cary 630 FT-IR (Agilent Technologies, Santa Clara, CA, USA) was applied to obtain the IR spectrum. Sephadex LH-20 (Merck), Silica gel (Merck, 230–400 mesh and 70–230 mesh, ASTM), and Diaion HP-20 (Mitsubishi, Tokyo, Japan) were used for open column chromatography. Pre-packed cartridges, Redi Sep-Silica (12 g, 24 g, 40 g, Teledyne Isco, Lincoln, NE, USA), and Redi Sep-C18 (13 g, 26 g, 43 g, 130 g, Teledyne Isco) were used for flash chromatography. Prep HPLC was performed using a Waters purification system (Waters corporation, MA, USA) equipped with a 1525 pump, a PDA 1996 detector, and a Luna NX-C18 100A column (250.0 × 21.2 mm i.d., 10.0 μm, Phenomenex, CA, USA).

### 3.2. Plant Material

The dried fruits of *Amomum tsao*-*ko* Crevost et Lemaire were purchased from Entaep Herb Co., Ltd. (Gyeonggi-do, Republic of Korea) in September 2020 and were identified by Professor Dae Sik Jang. A voucher specimen (AMTS-2020) was deposited in the herbarium of the College of Pharmacy, Kyung Hee University, Seoul, Republic of Korea.

### 3.3. Extraction and Isolation

The ground plant material (6.5 kg) was extracted with 25 L of acetone at room temperature over four days, and the extraction procedure was repeated three times. The extracts were filtered and then concentrated under reduced pressure at 45 °C. The acetone extract (157.2 g) was suspended in *n*-hexane and 90% methanol (MeOH), which yielded *n*-hexane- and MeOH-soluble fractions. The *n*-hexane-soluble fraction (85.5 g) was fractionated using silica gel (70–230 mesh; *ϕ* 6.5 × 42.0 cm) column chromatography (CC) with a gradient system [*n*-hexane/ethyl acetate (EtOAc) 98:2 to 50:50, *v*/*v*] to afford 21 fractions (H1~H21). Fraction H7 (11.0 g) was fractionated by Sephadex LH-20 CC [*ϕ* 4.6 × 68.0 cm, dichloromethane (DCM)] to obtain nine subfractions (H7-1~H7-9). Subfraction H7-8 (1.0 g) was separated by flash chromatography using a Redi Sep-RP cartridge (130g, MeOH/H_2_O = 70:30 to 90:10, *v*/*v*) to give compounds **8** (94.8 mg), **9** (11.4 mg), and **11** (145.2 mg). Fraction H8 (7.5 g) was subjected to Sephadex LH-20 CC (*ϕ* 4.6 × 62.0 cm, DCM/MeOH = 70:30, *v*/*v*) to afford five subfractions (H8-1~H8-5). Compound **7** (10.3 mg) was purified from subfraction H8-4 (708.4 mg) using a flash chromatographic system with a Redi Sep-RP cartridge (43 g, MeOH/H_2_O = 75:25 to 90:10, *v*/*v*). Fraction H9 (6.3 g) was separated by Sephadex LH-20 CC (*ϕ* 4.4 × 62.0 cm, DCM/MeOH = 70:30, *v*/*v*) to give four subfractions (H9-1~H9-4). Subfraction H9-3 (453.3 mg) was subjected to flash chromatography with a Redi Sep-RP cartridge (43 g, MeOH/H_2_O = 60:40 to 80:20, *v*/*v*) to obtain compound **12** (50.5 mg). Fraction H13 (14.7 g) was further fractionated by Sephadex LH-20 CC (*ϕ* 4.5 × 65 cm, DCM/MeOH = 0:100 to 50:50, *v*/*v*) to obtain four subfractions (H13-1~H13-4). Subfraction H13-2 (12.9 g) was chromatographed on silica gel (230–400 mesh; *ϕ* 4.7 × 31.0 cm) eluting with *n*-hexane/EtOAc (100:0 to 70:30, *v*/*v*), to give five subfractions (H13-2-1~H13-2-5). Subfraction H13-2-2 (7.4 g) was separated into yielding twelve subfractions (H13-2-2-1~H13-2-2-12) by reversed-phase CC (40–63 µm; *ϕ* 8.3 × 11.5 cm, acetone/H_2_O = 70:30 to 0:100, *v*/*v*). Subfraction H13-2-2-1 (30.9 mg) was purified by prep HPLC using a Luna NX-C18 100A column (250.0 × 21.2 mm i.d., 10.0 μm; acetonitrile/H_2_O = 50:50 to 70:30, *v*/*v*), yielding compound **6** (2.4 mg). Compound **4** (1.5 g) was isolated from fraction H13-2-2-2 (2.4 g) using a flash chromatographic system with a Redi Sep-RP cartridge (130g, acetonitrile/H_2_O = 60:40 to 75:25, *v*/*v*). Subfraction H13-2-4 (2.0 g) was subjected to reversed-phase CC (40–63 µm; *ϕ* 3.8 × 46.0 cm, acetone/H_2_O = 70:30 to 0:100, *v*/*v*) to obtain compound **10** (152.1 mg). Fraction H16 (2.3 g) was fractionated into four subfractions (H16-1~H16-4) by Sephadex LH-20 CC (*ϕ* 3.5 × 75.0 cm, DCM/MeOH = 0:100 to 50:50, *v*/*v*). Subfraction H16-3 (1.3 g) was separated further by flash CC with Redi Sep-silica cartridge (120 g, *n*-hexane/EtOAc = 100:0 to 20:80, *v*/*v*) to isolate compounds **1** (3.4 mg) and **3** (230.0 mg). Finally, fraction H20 (1.8 g) was chromatographed over Diaion HP-20 (*ϕ* 2.4 × 42.2 cm) eluting with MeOH-H_2_O (from 50:50 to 90:10, *v*/*v*) to afford five subfractions (H20-1~H20-5). Compounds **2** (1.2 mg) and **5** (29.0 mg) were isolated from subfraction H20-2 (102.8 mg) by flash CC with Redi Sep-RP cartridge (120 g, MeOH/H_2_O = 30:70 to 50:50, *v*/*v*.)

#### (*E*)-1-Acetyl-8-decene-1,10-diol (**1**, Tsaokol B)

Colorless oil; UV (acetonitrile) λ_max_ (log ε) 205 nm (2.24); IR (ATR) ν_max_ 3437, 2921, 2851, 1737, 1236 cm^−1^; HR-DART-MS (positive mode) *m/z* = 232.1900 [M+NH_4_]^+^ (calcd for C_12_H_26_N_1_O_3_ 232.1913); ^1^H-NMR (CDCl_3_, 500 MHz) and ^13^C-NMR (CDCl_3_, 125 MHz) data, see Table 1.

### 3.4. Synthesis of Tsaokol A

#### 3.4.1. Diethyl (2*E*,8*E*)-Deca-2,8-dienoate (Scheme 1)

A solution of cyclohexene (0.18 mL, 1.8 mmol) in DCM (4 mL) was cooled to −78 °C (dry ice, acetone) and ozone (from oxygen via generator) was bubbled into the solution until a blue color developed. Dry nitrogen gas was bubbled into the solution for several minutes to remove excess ozone. Triphenylphosphine (0.48 g, 1.8 mmol) was added to the cold solution followed by the direct addition of carbethoxymethylene triphenylphosphorane (1.5 g, 4.2 mmol), and the reaction mixture was allowed to warm to 25 °C for 16 h. The solvents were evaporated, and the crude residue (460 mg) was purified via Biotage Selekt with KP-SIL SNAP 50 g cartridge using EtOAc:*n*-hexane gradient 2–20% (flow rate 100 mL/min) to give 0.201 g (47% yield) of the diester as a clear oil.

**Scheme 1:**
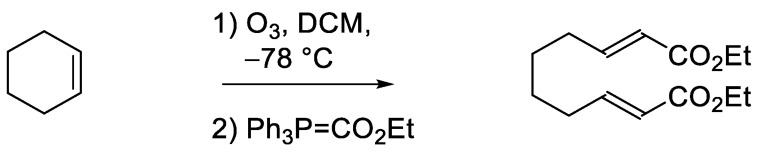
Synthesis of diethyl (*2E,8E*)-deca-2,8-dienoate.

^1^H NMR (CDCl_3_): 6.93 (2H, dt, *J* = 15.6, 6.8), 5.85 (2H, d, *J* = 15.6 Hz), 4.18 (4H, q, *J* = 7.2 Hz), 2.20–2.22 (4H, br d), 1.49 (4H, quin, *J* = 3.6 Hz), 1.29 (6H, t, *J* = 7.2 Hz).

#### 3.4.2. (2*E*,8*E*)-2,8-Decadiene-1,10-diol (**5**) (Scheme 2)

A solution of diethyl (2*E*,8*E*)-deca-2,8-dienoate (95 mg, 0.37 mmol) in DCM (3 mL) was cooled to −78 °C (dry ice, acetone). To this solution was added dropwise diisobutylaluminum hydride solution (1M in DCM, 1.5 mL, 1.5 mmol, 4 eq.) over 5 min. The reaction mixture was warmed to 0 °C and stirred for 2 h. This mixture was quenched by the addition of saturated aqueous potassium sodium tartrate (1 mL). The resultant solids were filtered through a bed of Celite and rinsed with DCM. The organic solvents were evaporated via Biotage V-10 to give 0.128 g crude clear oil. This residue was purified via Biotage Selekt with KP-Sil 10 g cartridge using EtOAc:*n*-hexane gradient 12–100% (flow rate 36 mL/min) to give 33 mg (52% yield) of **3** as a clear oil.

**Scheme 2:**
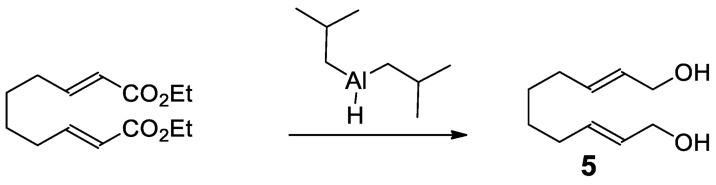
Synthesis of (2*E*,8*E*)-2,8-decadiene-1,10-diol (**5**).

#### 3.4.3. Tsaokol A (**3**) (Scheme 3)

To a 20 mL vial was added diol **5** (0.4 g, 2.3 mmol), DCM (6 mL), followed by triethylamine (0.65 mL, 4.7 mmol, 2 eq.) and acetic anhydride (0.22 mL, 2.3 mmol). This mixture was stirred at 25 °C for 16 h, at which time thin-layer chromatography (0.25 mm; EtOAc:*n*-hexane, 1:1) showed no starting material. The reaction mixture was evaporated via Biotage V-10, and the crude residue was purified/separated with Biotage Selekt via KP-Silica gel 25 g cartridge using EtOAc:*n*-hexane gradient 12–100% (flow rate 36 mL/min) to give two major products: diester **4** (240 mg oil) and mono-ester **3** (127 mg oil) (total 67% yield).

**Scheme 3:**
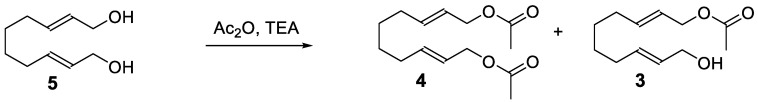
Synthesis of tsaokol A (**3**).

### 3.5. MIC Testing against M. tuberculosis

MICs against *M*. *tuberculosis* H37Rv (ATCC 27294) were determined as previously described using the Microplate Alamar Blue Assay (MABA) [23]. Briefly, 100x stocks of compounds in DMSO were prepared and 2 μL was transferred to a polystyrene transparent 96-well plate and compounds were serially diluted from columns 2–10. Column 11 contained bacteria without any drugs. An amount of 100 µL of *M*. *tuberculosis* H37Rv culture was added to each well. After 7 days of incubation at 37 °C, resazurin dye solution (20 μL of 0.6 mM resazurin dye and 12 μL of 20% Tween 80) was added to all the wells. After 24 h of incubation, fluorescence at 530 nm excitation and 590 nm emission was measured using a CLARIOstar (BMG LABTECH, Ortenberg, Germany) plate reader. The MIC was defined as the lowest concentration that reduced the fluorescence by 90% relative to the bacterial control. MICs of natural (**3**) and synthetic tsaokol A were the average of three biological replicates.

### 3.6. MIC Testing against Non-Replicating M. tuberculosis

MICs against non-replicating *M*. *tuberculosis* were determined as previously described by low oxygen recovery assay (LORA) [24], except by also using a recombinant auto-bioluminescent strain of *M*. *tuberculosis* H37Rv_LuxABCDE [25].

### 3.7. Cytotoxicity

Cytotoxicity against Vero cells (ATCC CRL-1586) was determined using a previously described method [26]. Vero cells were cultured in Eagle’s minimum essential medium (MEM) containing 10% FBS and penicillin and streptomycin. After verifying the morphology by microscopy, culture density was adjusted to 3 to 5 × 10^5^ cells/mL. An amount of 100 μL of the cell culture was inoculated on a 96-well plate with the test compounds for 72 h at 37 °C, 5% CO_2_. An amount of 20 μL of 0.6 mM resazurin dye was added to all of the wells and fluorescence at 530/590 nm (excitation/emission) was measured after 4 h of incubation. The IC_50_ was defined as the concentration of the test compound, causing a 50% reduction in fluorescence compared to the untreated cells.

## 4. Conclusions

Phytochemical investigation on the *n*-hexane-soluble fraction from the acetone extract of the fruits of *A*. *tsao*-*ko* led to the isolation of a new aliphatic compound **1** and a new natural aliphatic diester **6**, together with seven aliphatic compounds **2**–**5** and **7**–**9**, two acyclic monoterpenes **10** and **11**, and an acyclic sesquiterpene **12**. Among all the isolated compounds, isolated tsaokol A (**3**) and synthetic tsaokol A showed selective and potent in vitro growth inhibitory activity against *M*. *tuberculosis*. Further comprehensive in vitro profiling of tsaokol A (**3**) is currently underway by evaluating its activity against intracellular *M*. *tuberculosis* and non-tuberculous mycobacteria. Isolation of resistant mutants against the tsaokol A (**3**) is also ongoing to possibly deconvolute the target protein and the mode of action.

## Figures and Tables

**Table 1:** ^1^H and ^13^C NMR assignments for compounds **1** and **3** (*δ* in ppm, CDCl_3_, 500 and 125 MHz).

Position ^a^	1		3	
	*δ*_H_ Multi (*J* in Hz)	*δ*_c_	*δ*_H_ Multi (*J* in Hz)	*δ*_c_
1	4.03 t (7.0)	64.8	4.49 dd (6.5, 1.0)	65.4
2	1.59 m	28.7	5.55 dt (15.0, 6.5)	124.0
3	1.20–1.39 ^b^	26.0	5.74 dtt (15.0, 6.5, 1.0)	136.5
4		29.3	2.04 m ^b^	32.1
5		29.9	1.38 m ^b^	28.7
6		29.2	1.38 m ^b^	28.4
7	2.02 m ^b^	32.4	2.04 m ^b^	32.2
8	5.67 dt (15.5, 6.5)	133.6	5.66 dt (15.0, 6.5)	133.2
9	5.61 dt (15.5, 5.5)	129.1	5.63 dt (15.0, 5.0)	129.2
10	4.07 d (5.5)	64.1	4.07 d (5.0)	63.9
1-OCOCH_3_		171.5		171.1
1-OCOCH_3_	2.02 s ^b^	21.2	2.05 s ^b^	21.2

^a^ Determined by analysis of ^1^H-^1^H COSY, ^1^H-^13^C HSQC, and HMBC spectrum. ^b^ Overlapped.

**Table 2:** Activity of compounds **1**–**12** isolated from the fruits of *A*. *tsao*-*ko* and synthetic Tsaokol A against replicating (MABA) and non-replicating *M*. *tuberculosis* H37Rv (LORA).

Extract or Compounds		MIC (µg/mL) against *M*. *tuberculosis* H37Rv		Cytotoxicity against Vero Cells
		MABA	LORA	IC_50_
-	Acetone extract	9.7	86.8	92.8
**1**	Tsaokol B	10.2	61.5	>200 (10%)
**2**	(*E*)-2-Decene-1,10-diol	29.7	95.4	>200 (25%)
**3**	Tsaokol A	0.6	1.4	>200 (7%)
**4**	Acetoxytsaokol A	1.1	21.6	143.6
**5**	(2*E*,8*E*)-2,8-Decadiene-1,10-diol	4.3	5.9	72.9
**6**	(2*E*,6*E*)-1,8-Diacetoxy-2,6-octadiene	26.1	>100 (49%)	76.8
**7**	(*E*)-Decenal	>100 (68%)	>100 (67%)	
**8**	(*E*)-Dec 2-enyl acetate	>100 (48%)	>100 (14%)	
**9**	(*E*)-2-Dodecen-1-yl acetate	>100 (81%)	>100 (50%)	
**10**	Geraniol	>100 (16%)	>100 (16%)	
**11**	Geranyl acetate	88.6	>100 (8%)	>200 (21%)
**12**	(3*R*)-(*E*)-Nerolidol	>100 (71%)	>100 (71%)	
	Synthetic tsaokol A	1.4		>200 (10%)
	Rifampicin	0.025	0.14	>100 (50%)
	Isoniazid	0.03	>18 (85%)	
	Linezolid	0.16	0.62	

## Data Availability

Not applicable.

## Supplementary Materials

The following supporting information can be downloaded at: https://www.mdpi.com/article/10.3390/plants12010034/s1. Figure S1. DART-MS spectrum of compound **1**.; Figure S2. The IR spectrum of compound **1**.; Figure S3. The ^1^H-NMR (500 MHz, CDCl_3_) spectrum of compound **1**.; Figure S4. The ^13^C-NMR (125 MHz, CDCl_3_) spectrum of compound **1**.; Figure S5. The HSQC spectrum of compound **1** in CDCl_3_.; Figure S6. The COSY spectrum of compound **1** in CDCl_3_.; Figure S7. The HMBC spectrum of compound **1** in CDCl_3_.; Figure S8. DART-MS spectrum of compound **6**.; Figure S9. The IR spectrum of compound **6**.; Figure S10. The ^1^H-NMR (500 MHz, CDCl_3_) spectrum of compound **6**.; Figure S11. The ^13^C-NMR (125 MHz, CDCl_3_) spectrum of compound **6**.
