# Supplementary material for: Anti-Microbial Activity of Aliphatic Alcohols from Chinese Black Cardamom (Amomum tsao-ko) against Mycobacterium tuberculosis H37Rv
Source: Plants (Basel). 2022 Dec 21;12(1):34. doi: 10.3390/plants12010034 (PMC9823811; doi:10.3390/plants12010034)

Supporting information

Anti-microbial Activity of Aliphatic Alcohols from Chinese Black Cardamom (*Amomum tsao-ko*) against *Mycobacterium tuberculosis* H37Rv

So Young Lee [1,†], Gauri S. Shetye [2,†], So-Ri Son [1], Larry L. Klein [2], Jeffrey K. Yoshihara [2], Scott G. Franzblau [2], Sanghyun Cho [b,*], Dae Sik Jang [1, 3,*]

Contents of supporting information

Figure S1. DART-MS spectrum of compound **1**.

Figure S2. The IR spectrum of compound **1**.

Figure S3. The ^{1}H-NMR (500 MHz, $CDCl_3$) spectrum of compound **1**.

Figure S4. The ^{13}C-NMR (125 MHz, $CDCl_3$) spectrum of compound **1**.

Figure S5. The HSQC spectrum of compound **1** in $CDCl_3$.

Figure S6. The COSY spectrum of compound **1** in $CDCl_3$.

Figure S7. The HMBC spectrum of compound **1** in $CDCl_3$

Figure S8. DART-MS spectrum of compound **6**.

Figure S9. The IR spectrum of compound **6**.

Figure S10. The ^{1}H-NMR (500 MHz, $CDCl_3$) spectrum of compound **6**.

Figure S11. The ^{13}C-NMR (125 MHz, $CDCl_3$) spectrum of compound **6**.

Figure S1. DART-MS spectrum of compound **1**

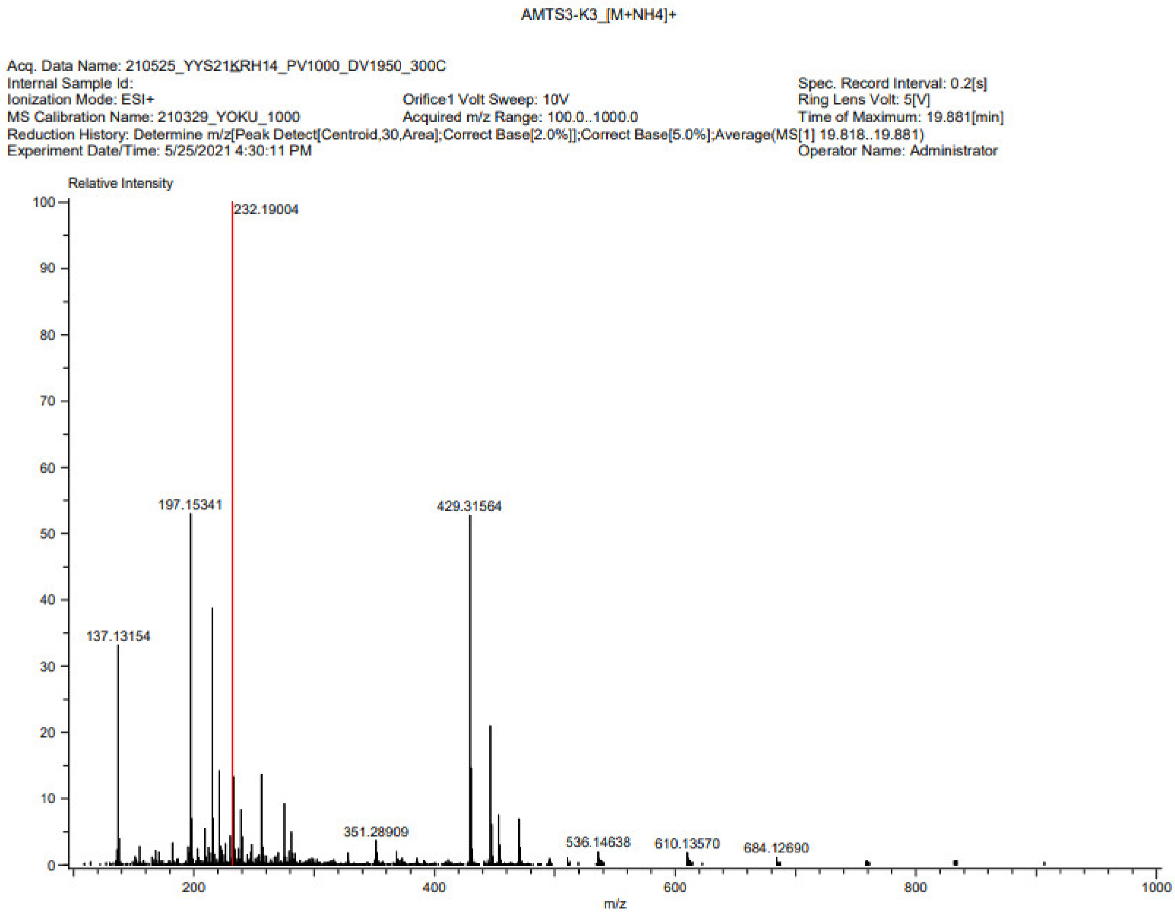

Figure S2. The IR spectrum of compound **1**.

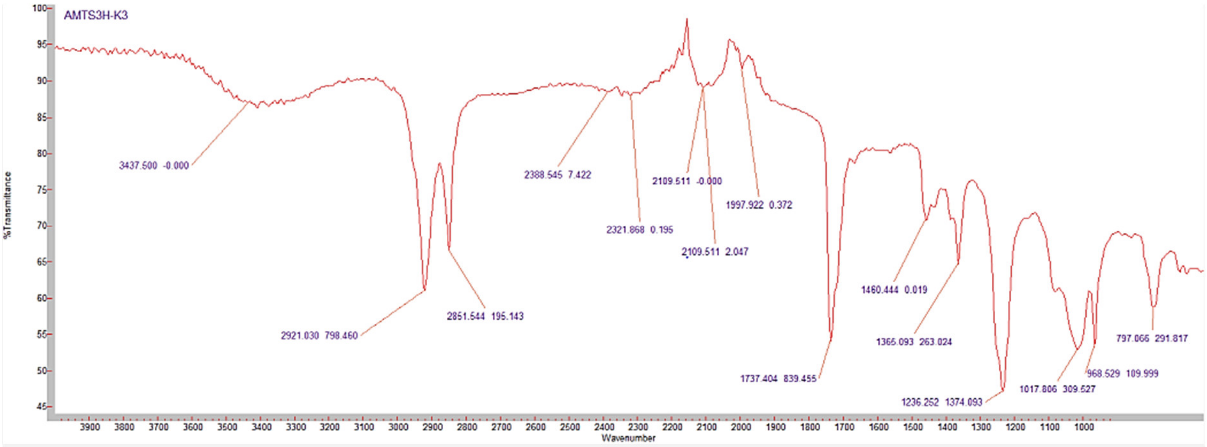

Figure S3. The ^{1}H-NMR (500 MHz, $CDCl_3$) spectrum of compound **1**

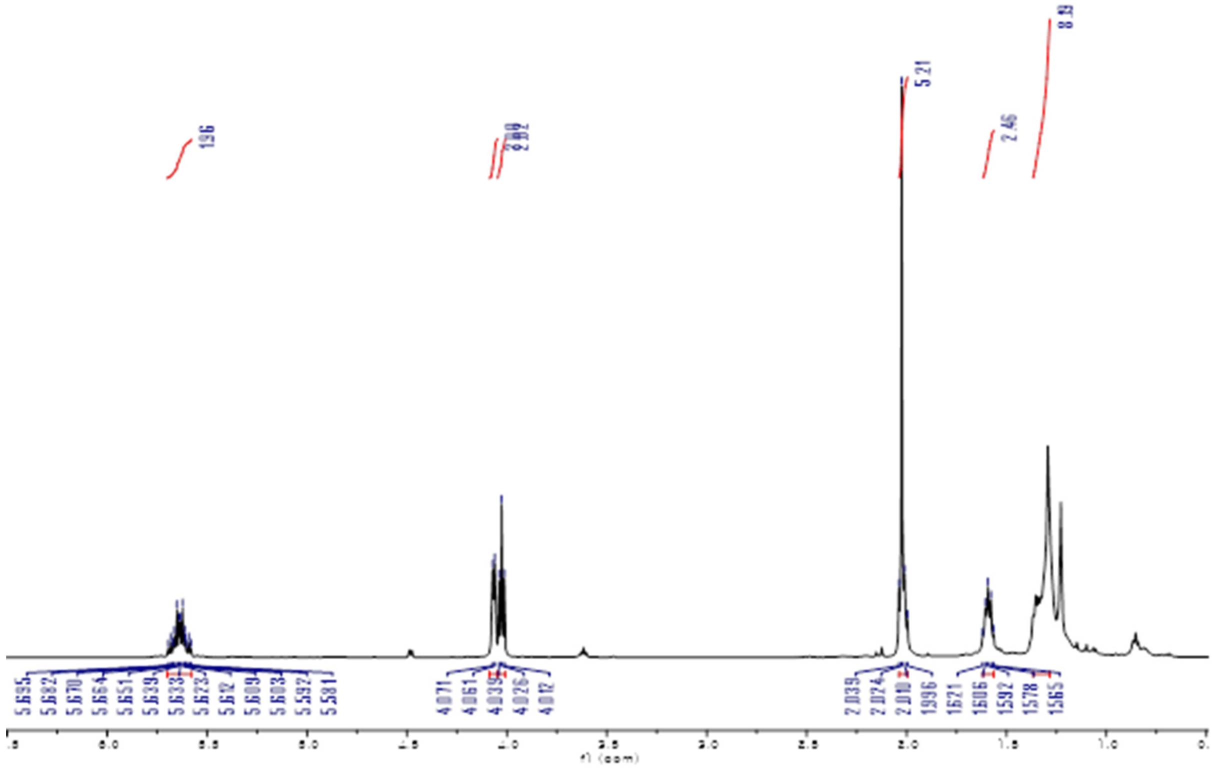

Figure S4 The ^{13}C-NMR (125 MHz, $CDCl_3$) spectrum of compound **1**.

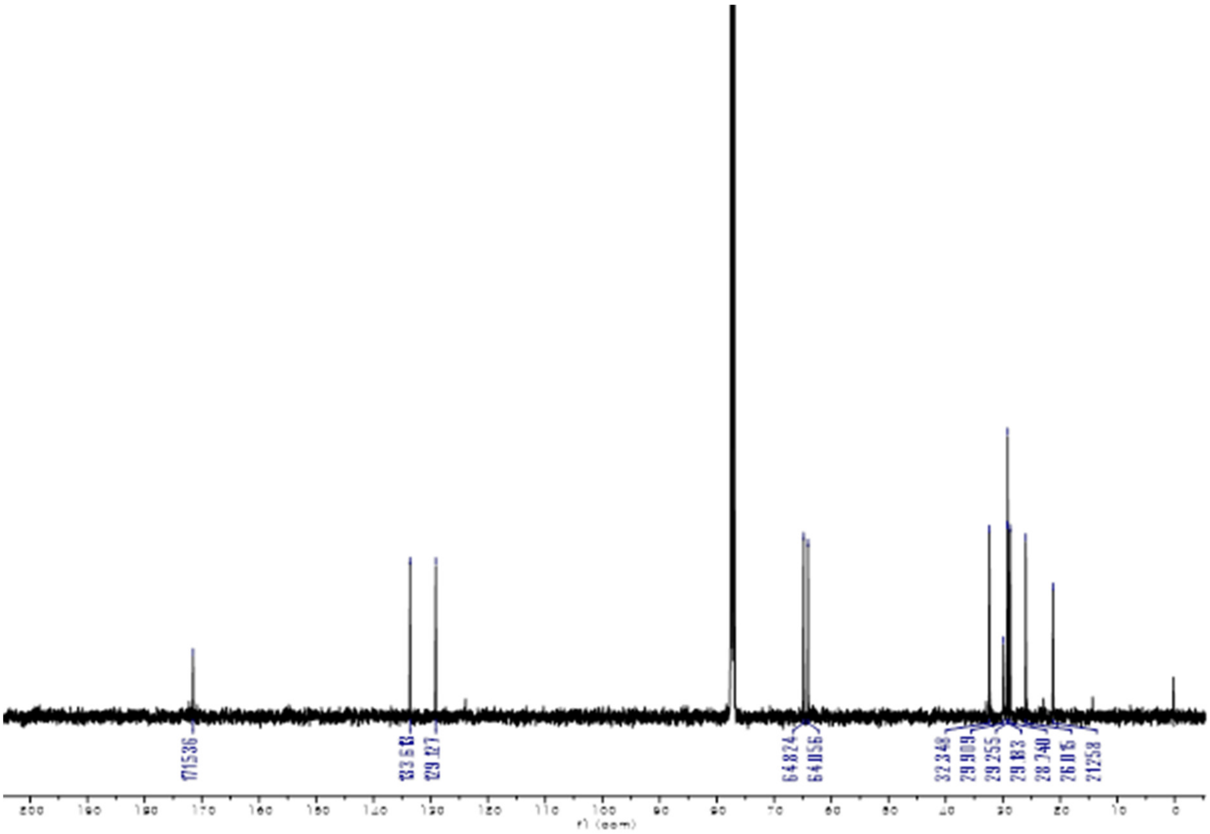

Figure S5. The HSQC spectrum of compound **1** in $CDCl_3$.

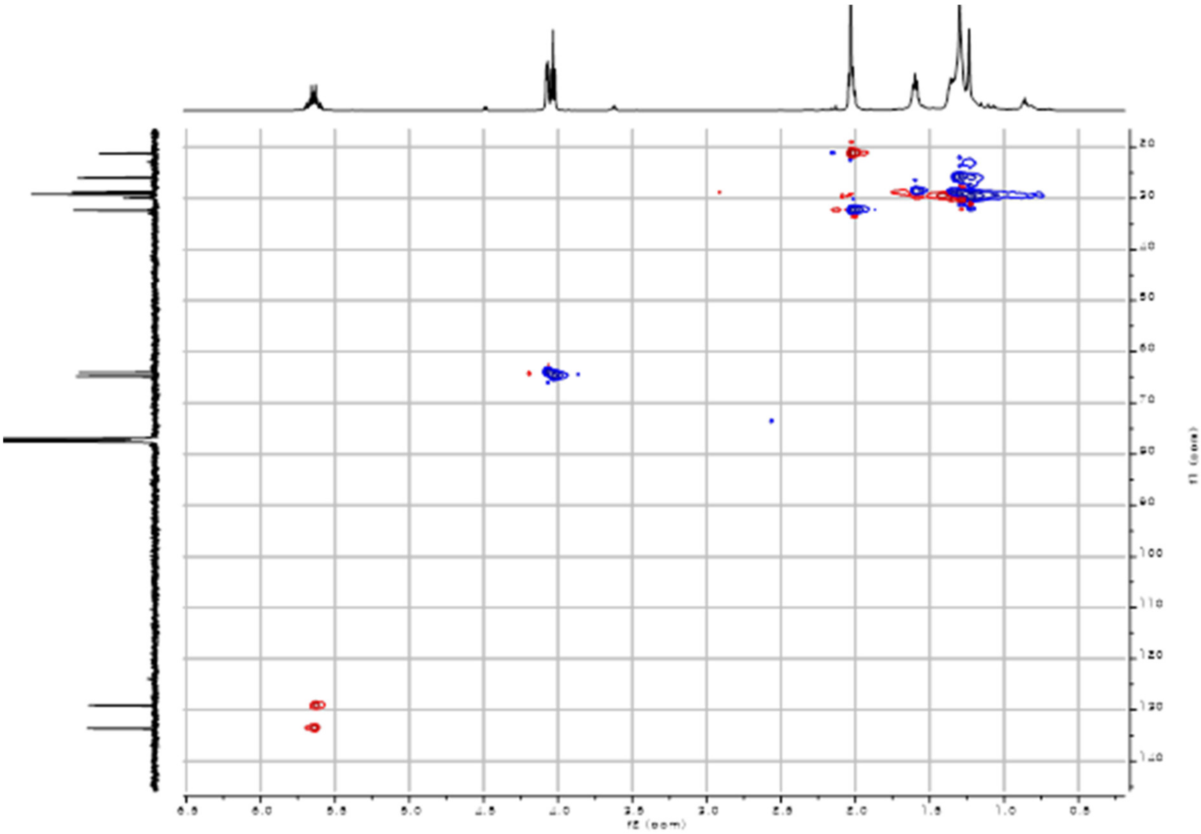

Figure S6. The COSY spectrum of compound **1** in $CDCl_3$.

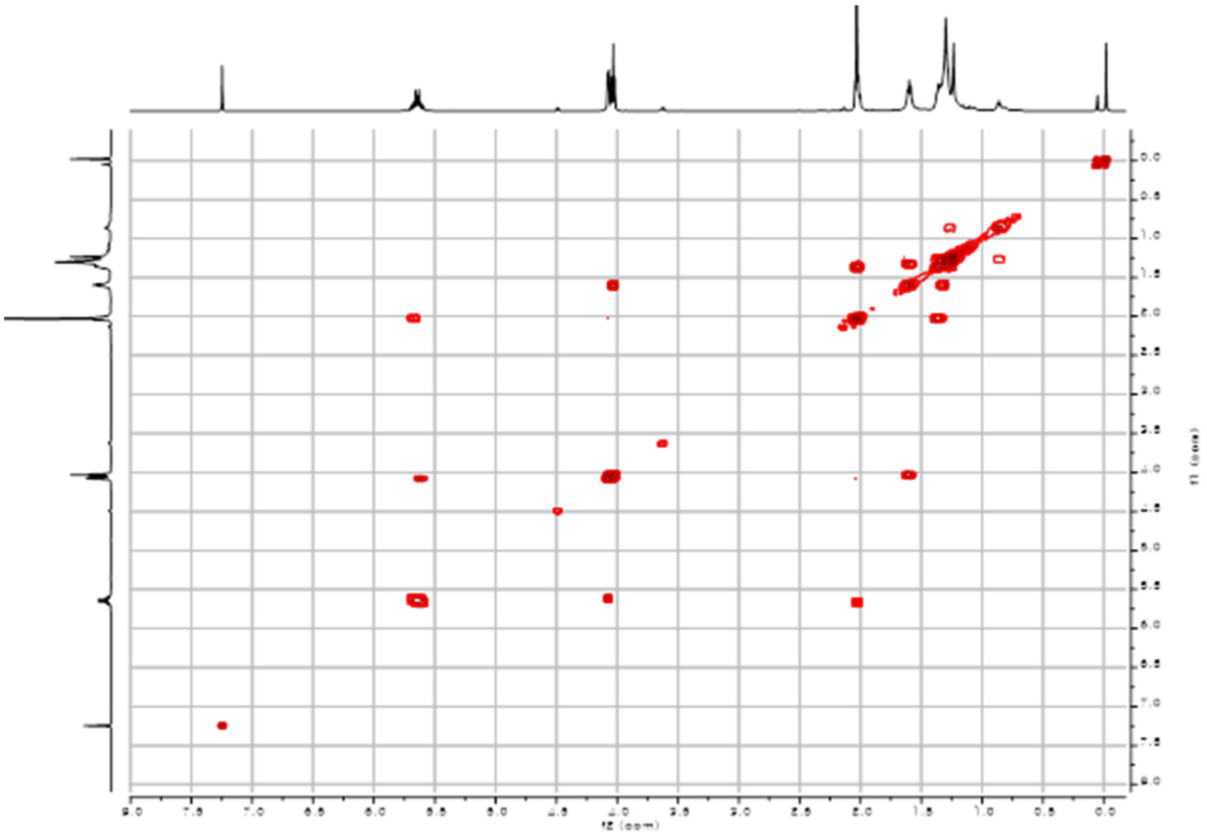

Figure S7. The HMBC spectrum of compound **1** in $CDCl_3$.

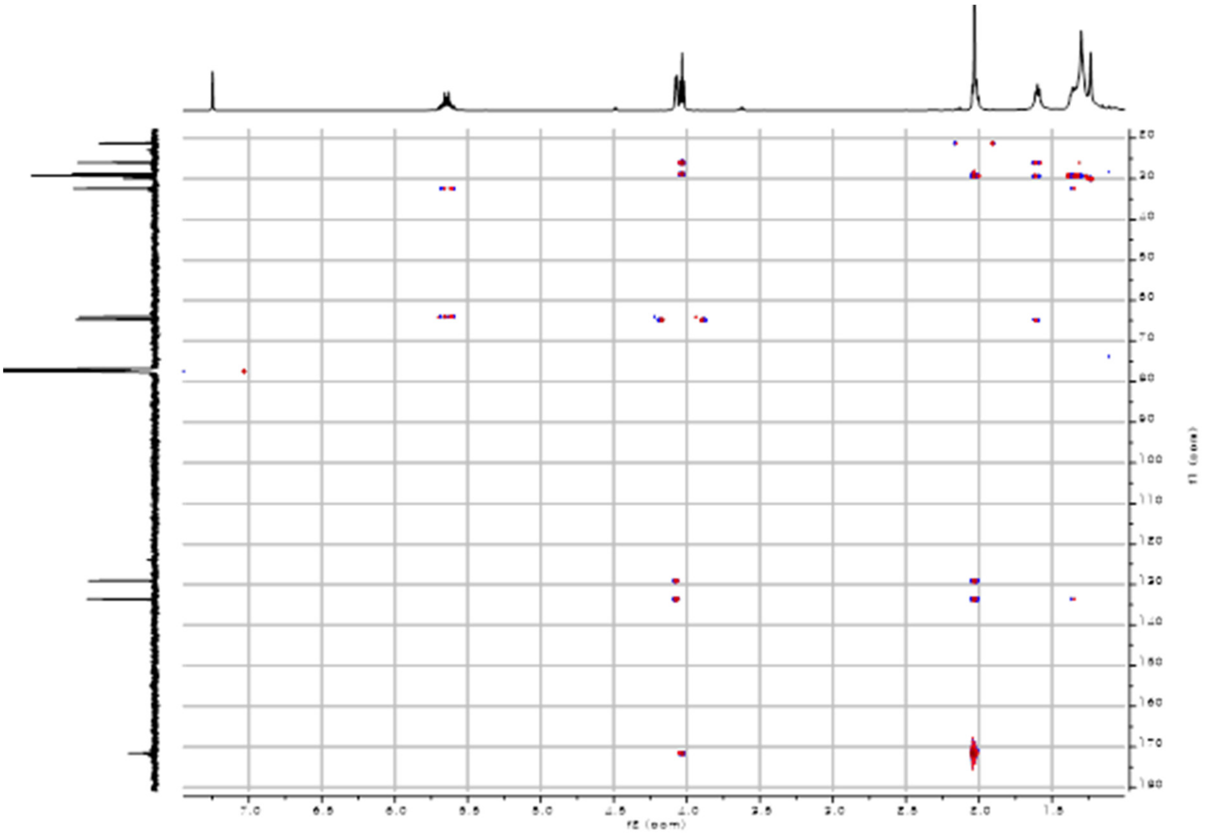

Figure S8. DART-MS spectrum of compound **6**.

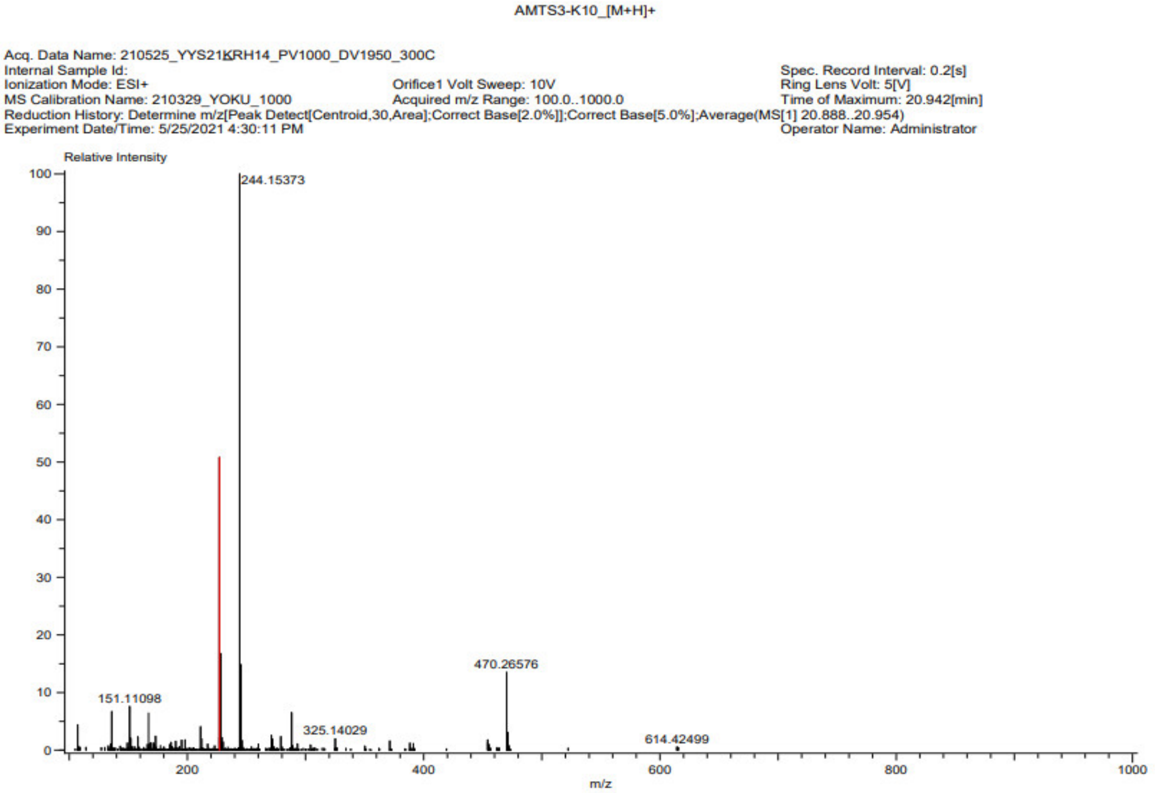

Figure S9. The IR spectrum of compound **6**.

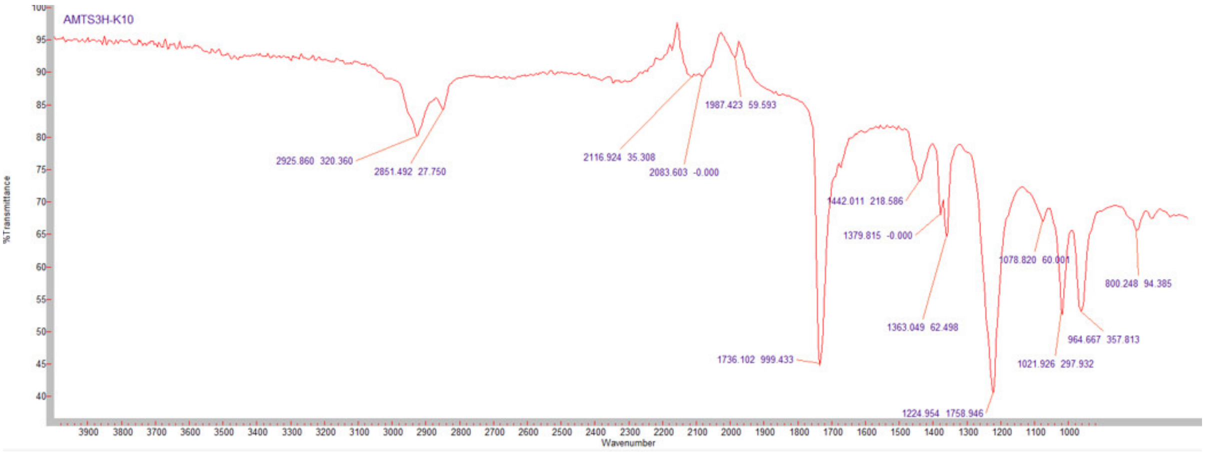

Figure S10. The ^{1}H-NMR (500 MHz, $CDCl_3$) spectrum of compound **6**.

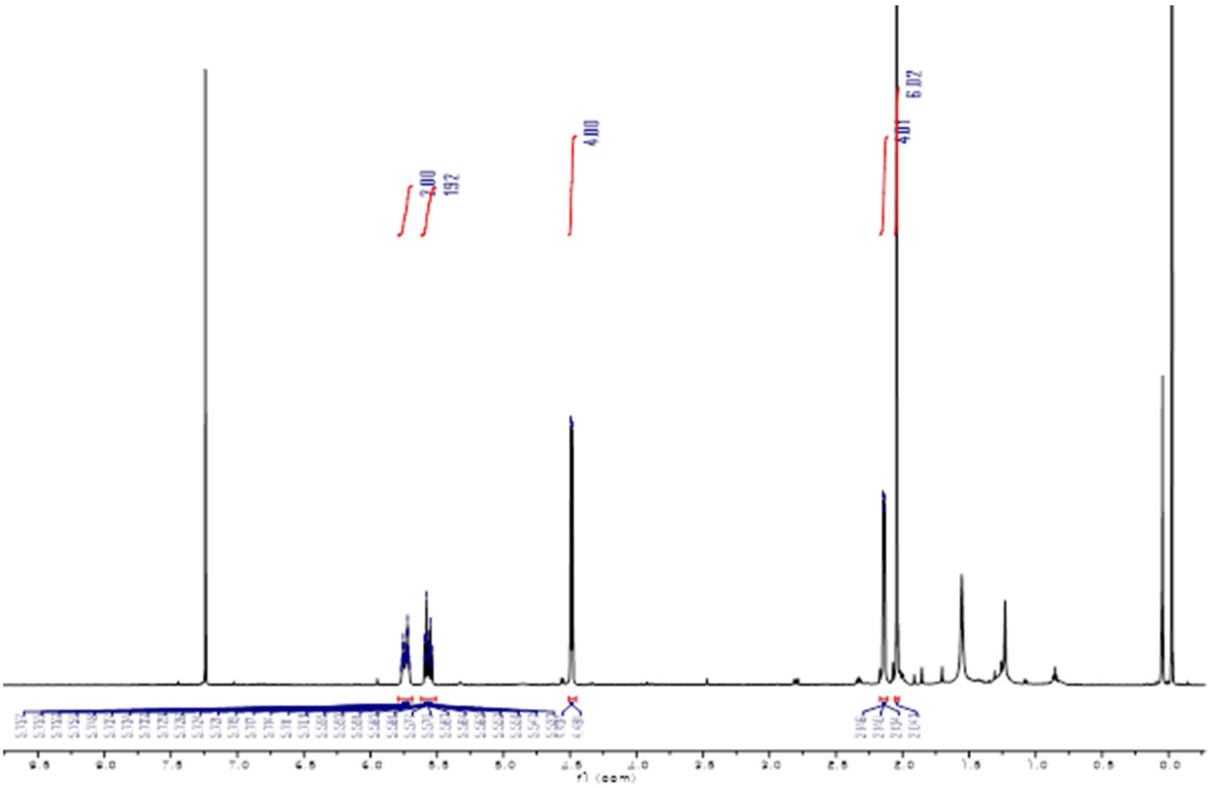

Figure S11. The ^{13}C-NMR (125 MHz, $CDCl_3$) spectrum of compound **6**.

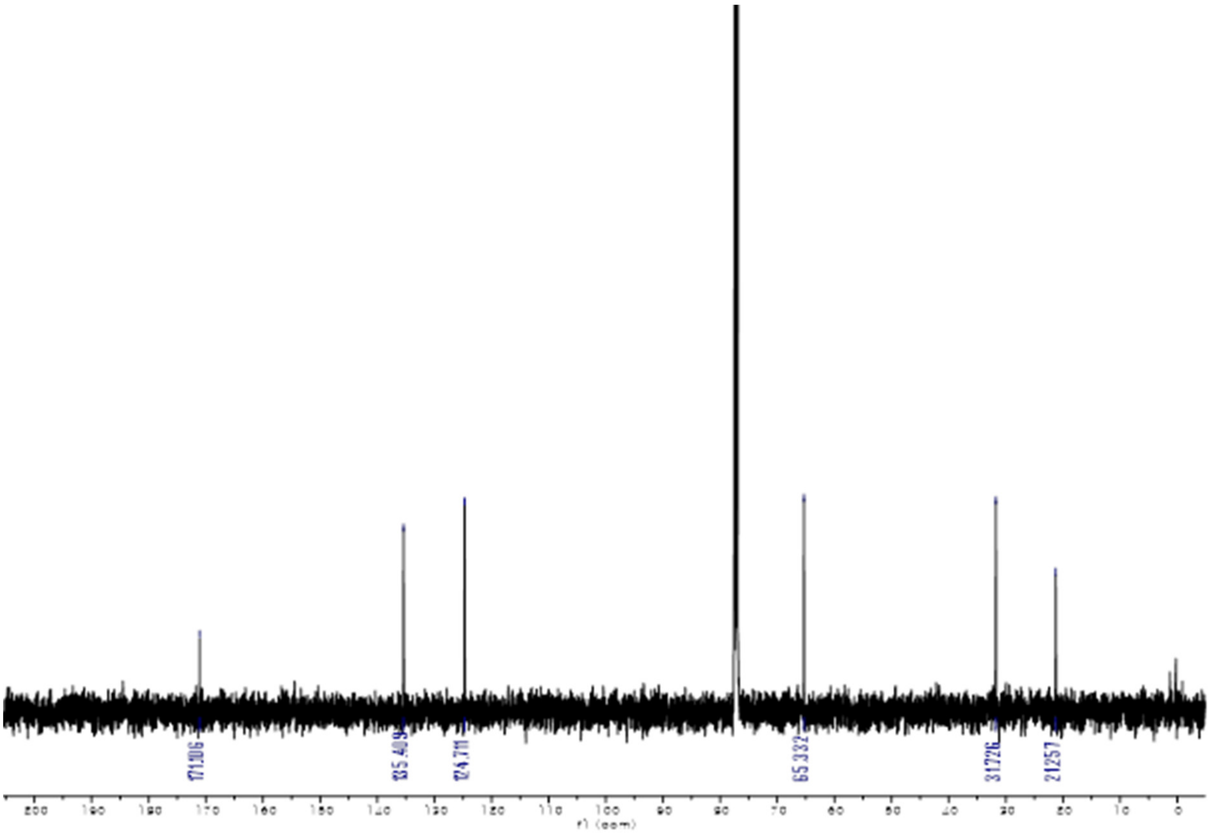

Supplement: Supplementary file 1 [file plants-12-00034-s001.zip › plants-2093207-supplementary.pdf]
